# Sonic Hedgehog Is a Chemoattractant for Midbrain Dopaminergic Axons

**DOI:** 10.1371/journal.pone.0007007

**Published:** 2009-09-23

**Authors:** Rachel Hammond, Sandra Blaess, Asa Abeliovich

**Affiliations:** 1 Departments of Pathology and Neurology, Center for Neurobiology and Behavior and Taub Institute, Columbia University College of Physicians and Surgeons, New York, New York, United States of America; 2 Developmental Biology Program, Sloan-Kettering Institute, New York, New York, United States of America; 3 Institute of Reconstructive Neurobiology, Life and Brain Center, University of Bonn, Bonn, Germany; Harvard University, United States of America

## Abstract

Midbrain dopaminergic axons project from the substantia nigra (SN) and the ventral tegmental area (VTA) to rostral target tissues, including the striatum, pallidum, and hypothalamus. The axons from the medially located VTA project primarily to more medial target tissues in the forebrain, whereas the more lateral SN axons project to lateral targets including the dorsolateral striatum. This structural diversity underlies the distinct functions of these pathways. Although a number of guidance cues have been implicated in the formation of the distinct axonal projections of the SN and VTA, the molecular basis of their diversity remains unclear. Here we investigate the molecular basis of structural diversity in mDN axonal projections. We find that Sonic Hedgehog (Shh) is expressed at a choice point in the course of the rostral dopaminergic projections. Furthermore, in midbrain explants, dopaminergic projections are attracted to a Shh source. Finally, in mice in which Shh signaling is inactivated during late neuronal development, the most medial dopaminergic projections are deficient.

In addition to the role of Shh in the induction of mDN precursors, Shh plays an important role in dopaminergic axon pathfinding to rostral target tissues. Furthermore, Shh signaling is involved in determining the structural diversity of these dopaminergic projections.

## Introduction

Midbrain dopaminergic neurons (mDN) play essential roles in diverse mammalian behaviors, including motor control and reward-associated learning [1], [2]. This functional diversity is a consequence of structural heterogeneity in mDN axonal projections.

**Figure 1:**
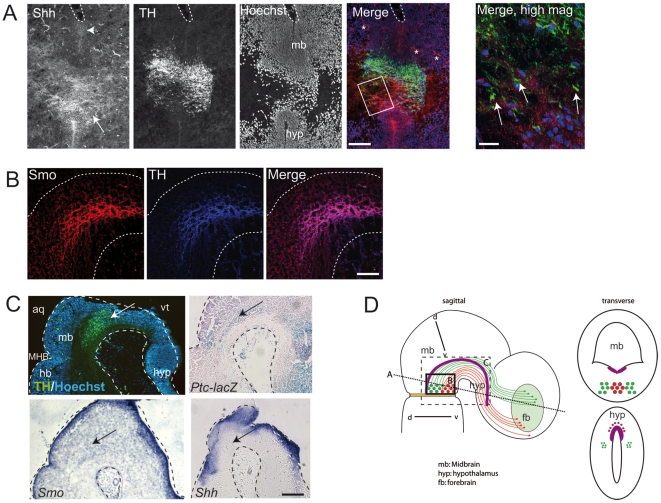
Sonic Hedgehog signaling is a candidate guidance cue for mDNs. (A, B) Immunohistochemical analysis of Sonic Hedgehog (Shh) and Smoothened (Smo) expression in relation to the tyrosine hydroxylase (TH)- positive midbrain dopaminergic neurons (mDN) in E12.5 mouse embryos. (A) Shh expression in the ventral midline of the midbrain (mb) ventricular zone (indicated by arrowhead in left panel) lies dorsal to differentiated mDN (TH+). Shh expression in the hypothalamus (hyp) lies ventral and rostral to the dopaminergic cell bodies (indicated by arrow in left panel). Note that TH+ mDN axonal projections are found in the Shh expressing region in the hypothalamus (arrows in right panel). The right panel, a higher magnification of the boxed area in the merged image, is a maximum intensity projection of a Z-stack acquired with the Zeiss ApoTome system. Asterisks in merged image indicate background staining of blood vessels. Aqueduct is outlined. Immunostaining was performed on coronal sections. The plane of section is indicated in schematic in D. (B) Smo expression co-localizes with the midbrain dopaminergic population. Immunostaining was performed on sagittal sections. The area shown is indicated in the schematic in D. (C) Mid-sagittal sections of E12.5 embryonic brain. Arrows indicate location of dopaminergic neurons. The area shown is indicated in the schematic in D. TH/Hoechst: Immunostaining for TH and Hoechst showing the location of dopaminergic neurons (arrow) in relation to the aqueduct (aq) and 3^rd^ ventricle (vt) (outlined) and the mid/hindbrain boundary (MHB). Ptc: Analysis of *Ptc-lacZ* [18] mice by X-gal staining for β-galactosidase activity shows expression of Patched in the ventral midbrain. Blue staining indicates Ptc-lacZ expression, pink staining is a nuclear stain (nuclear fast red). Smo: In situ hybridization for Smo shows expression of Smo in the ventral midbrain. Shh: In situ hybridization for Shh shows expression dorsal (in mb) and rostral (in hyp) to the mDN cell population. (D) Schematic of sagittal (left panel) and transverse sections (right panels) of midbrain at E12.5. Lateral SN neuronal projections that course to the dorsolateral striatum precursor are in green. Medial ventral projections from the VTA, which project to the nucleus accumbens precursor, are in red. Additional medial fibers project ventrally to the pallidum. Pink line depicts the floor plate and ventral midline. Sagittal schematic: Brown line displays the midbrain-hindbrain junction. Dashed line corresponds to the orientation of the transverse section in A. Black box depicts the sagittal sections in B. Dashed box depict sagittal sections in C. Transverse schematic: Green and red circles in midbrain depict mDN cell bodies, small green and red circles in hypothalamus depict mDN axonal projections. Scale bars: (A) 100 µm (merge) and 20 µm (high mag); (B) 50 µm; (C) 200 µm.

Mammalian mDN axonal projections course rostrally towards forebrain structures that are expanded in both the dorsoventral and mediolateral axes. The most medial and ventral mDN projections, within the VTA, regulate reward-associated behavior and novelty learning and project rostrally on a ventromedial course to the nucleus accumbens and to extrastriatal structures including the pallidum and subthalamus. In contrast, more lateral mDN axons originate within the SN and take a relatively dorsolateral course on their rostral traverse (Figure 1A). Although several factors, including Slit-1 and -2 [3], Netrins [4], Ephrins [5] and Semaphorins [6], have been implicated in mDN pathfinding, the molecular basis of the diversity of mDN axonal projections is unknown.

In this study, we identify Shh as an axonal chemoattractant for mDN axons both in vitro and in vivo. Shh is essential for many aspects of early mammalian development [7] and plays a central role in ventral patterning along the entire neuroaxis at early developmental stages (E7.5–E9.5). Additionally, Shh has been shown to function as a chemoattractant for ventrally and rostrally coursing spinal cord commissural neurons [8], [9]. In the embryonic midbrain, Shh is essential for the specification of mDNs, but only prior to embryonic day (E) 10 [10], [11]. We show here that Shh acts as an axonal guidance cue for dopaminergic axons after dopaminergic neuron specification is completed. Furthermore, we present evidence that Shh signaling plays a role in determining the diversity of mDN axonal projections.

## Results

In a screen of potential guidance cues that are expressed at the midline along the rostral course of mDN axons and temporally coincide with their passage (at E12.5–E15.5 in the mouse), we identified Shh as a candidate. Shh is expressed in the ventral midline (floor plate) at this time point in development, as demonstrated both by immunohistochemistry for Shh and in situ hybridization for *Shh* mRNA transcript (Figures 1A and 1C). Prior studies have established the presence of a Shh gradient from ventral to dorsal regions throughout the neuroaxis, including the midbrain [12]. mDN axons, identified by the expression of tyrosine hydroxylase (TH, the rate-limiting enzyme of dopamine synthesis), express the Shh receptor component Smoothened (Smo; Figure 1B, 1C). Additionally, the Shh receptor component, Patched (Ptc), is expressed in the ventral midbrain region where the mDNn are located (Figure 1C). Interestingly, other canonical Shh signaling components including Gli1, Gli2, and Gli3 [11], [13]; (data not shown), are not expressed in the mDNs at this stage of embryogenesis.

**Figure 2:**
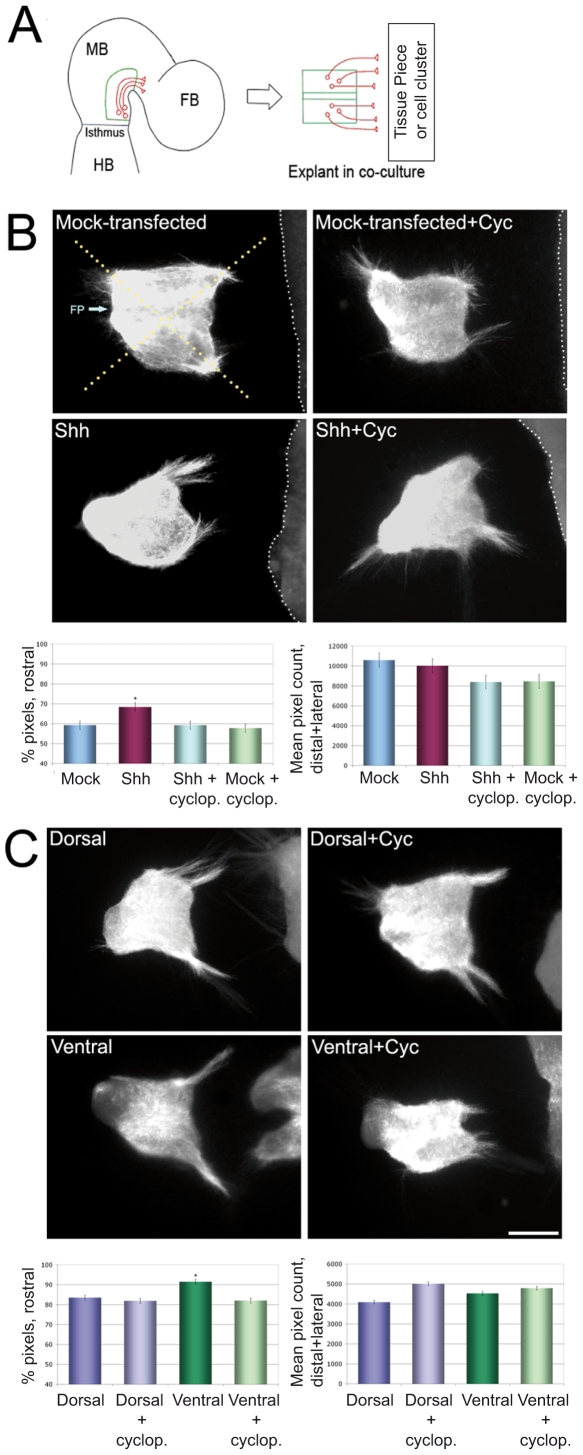
Shh as a chemoattractant in mDN explant cultures. (A) Schematic of explant dissection. The ventral third of the midbrain neuroepithelium, including the floor plate, of E11.5 mouse was dissected and placed in an ‘open-book’ preparation in apposition to transfected HEK293T cells or appropriate tissue as indicated. HB – hindbrain, MB – midbrain, FB – forebrain. (B) E11.5 bilateral ventral midbrain explants cultures (FP – floor plate) in apposition to mock-transfected or Shh transfected HEK293T cells (delineated by white dotted lines). Explants and cell clusters were cocultured for 3 days, fixed, and immunostained for TH. Explants demonstrated increased TH-positive axonal outgrowth in the rostral quadrant (facing the explant – quadrants delineated by yellow dotted lines) in the presence of a Shh source. This effect was diminished by the addition of cyclopamine (Cyc) at 10 nM to the culture medium. The axonal extension from the rostral side was quantified using pixel counting software (see Materials and Methods) and expressed as a percentage of total rostral and caudal outgrowth. The effect of Shh was significant when tested using the two-tailed Student T-test (p<0.05), n>25 explants in each condition. Total (non-directional) outgrowth from the lateral and caudal sides remained unaffected by the presence of Shh. (C) The effects of Shh transfected HEK293T cells can be mimicked by the presence of rostro-ventral neuroepithelium known to express Shh (excluding the zona limitans intrathalamica; ZLI), and this effect can be reduced by the addition of cyclopamine to the medium. This effect is significant (p<0.05) by the two-tailed Student T-test, n>25 explants in each condition. Total axonal outgrowth from the caudal and lateral sides of the explants appears unaffected by the presence of dorsal or ventral tissue pieces. However dorsal tissue, which lacks Shh expression, does appear to have a positive effect on dopaminergic axonal outgrowth from the rostral side of ventral midbrain explants, an effect that is not blocked by cyclopamine implicating a Shh/Smo independent mechanism. Scale bar: (B+C) 100 µm.

To investigate a role for Shh as a chemoattractant for mDN axons, we established an explant culture model in which E11.5 midbrain tissue was maintained next to an aggregate of HEK293 cells transfected with full-length Shh cDNA or control vector for 3 days (Figure 2A). Whereas control vector-transfected HEK293 had no effect, cells expressing Shh promoted unidirectional outgrowth toward the Shh source of TH-positive dopaminergic axons (Figure 2B). If Shh is a chemoattractant for TH-positive neurons, we would predict that ventral midline tissue rostral to the explant, which expresses Shh (Figure 1A, 1C), should also attract mDN axons. Consistent with this, ventral (but not dorsal) tissue was effective at attracting mDN axons (Figure 2C). Cyclopamine, a specific antagonist of Smo function, partially blocked the chemoattractant activity of ventral tissue, indicating a role for Shh as a ventral guidance cue for mDN axons. The remaining attractant activity of the ventral tissue is likely due to an additional, unidentified chemoattractant expressed in the rostral ventral areas. Shh did not alter the overall outgrowth of TH-positive axons from the explant, but rather promoted unidirectional outgrowth towards the chemoattractant (Figure 2B). As residual endogenous Shh may be present in the midbrain explants, the effects we observe may underestimate the role of Shh. E11.5 midbrain explants harbor mature mDNs, and consistent with this, Shh did not alter the number or specification of mDNs in the explants (data not shown).

**Figure 3:**
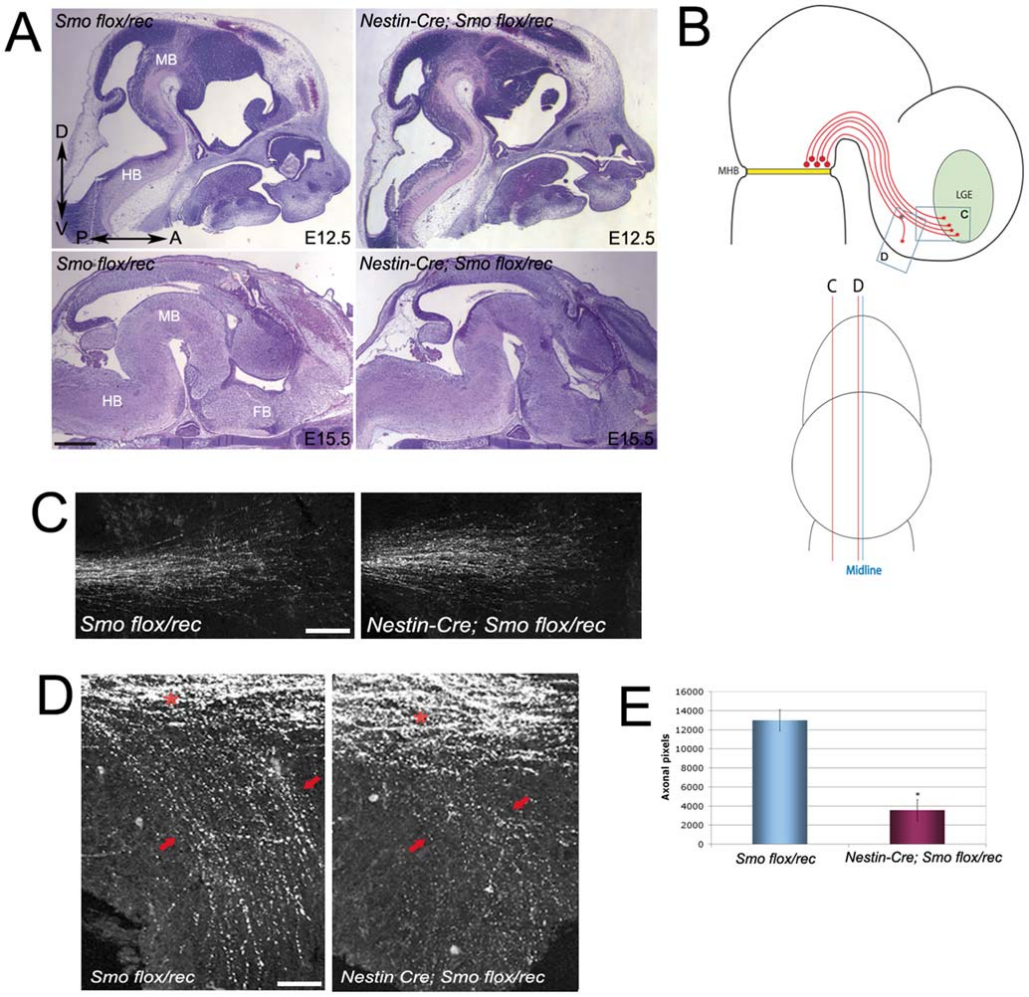
Smoothened conditional mutant mice display aberrant mDN axonal projections. (A) Hematoxylin and eosin staining on sagittal sections of *Nestin-Smo* cko mice (*Nestin-Cre; Smo ^flox^/^rec^*) mutant mice and their control (*Smo ^flox^/^rec^*) littermates at E12.5 and E15.5. The histology of the conditional mutants closely resembles that of their *smo^flox^/smo^rec^* littermates. HB – hindbrain, MB – midbrain, FB – forebrain; A – anterior, P – posterior, D – dorsal, V – ventral. (B) Schematics illustrating the axonal projections of the midbrain dopaminergic neurons (red), which project rostrally away from the midbrain-hindbrain boundary (MHB). The majority of these neurons project towards the lateral ganglionic eminence (LGE), the striatal precursor, but a subset constitutes the extra-striatal projection and courses more ventrally. The regions imaged in panels C and D of this figure are schematized by grey boxes in the first schematic, and their medio-lateral position in the second. (C) The dopaminergic (TH positive) projections to the LGE are comparable between *Nestin-Smo* cko mice and *control* mice at E13.5, which is confirmed by pixel counting across lateral sections (n = 4 mice in each, data not shown). (D) In control mice the extra-striatal dopaminergic projections course ventrally, but in *Nestin-Smo* cko mice these projections are much reduced, and some axons appear to misdirected. Asterisk indicates the ‘choice point’ where axons either project rostrally towards the LGE, or project ventrally to form the extra-striatal projection (indicated by red arrows). (E) Quantification of the reduction in extra-striatal projection, pixels counted across 6 medial most sections in n = 4 animals for each condition. Statistical significance assessed using 2-tailed Student T-test (p<0.01). Scale bars: (A) 500 µm, (B+C) 100 µm.

**Figure 4:**
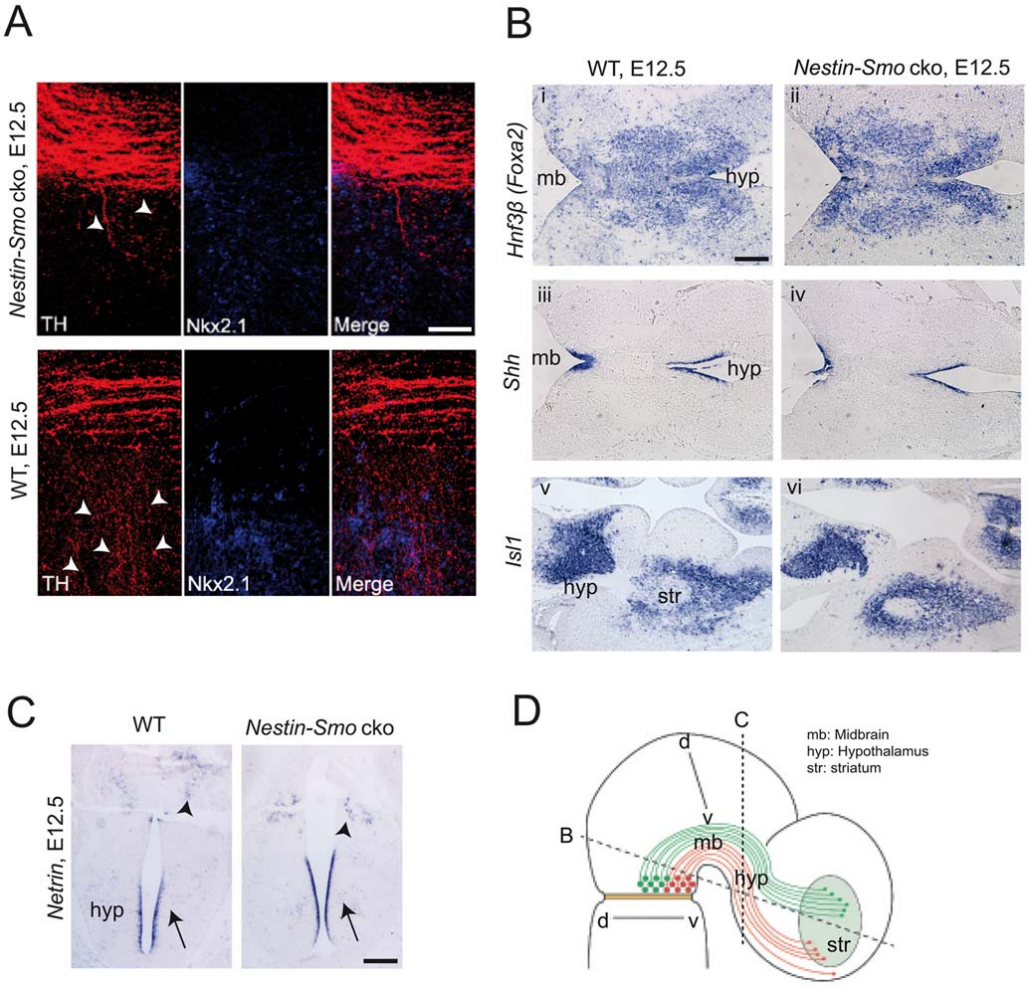
Dopaminergic projection alterations in Smoothened conditional mutant mice do not appear to be a consequence of target tissue defects. (A) At the choice point for rostrally coursing mDN axonal projections (as in Figure 3) the most medial fibers course ventrally (arrowheads in lower left panel) to the embryonic pallidum, subthalamus, and hypothalamus, as indicated by immunostaining with an antibody specific for Nkx2.1. These fibers are deficient in the *Nestin-Smo* cko mice at E12.5 (arrowheads in upper left panel). (B) Analysis of gene expression by in situ hybridization in the midbrain and target tissues of wild-type and *Nestin-Smo* cko mice at E12.5. Expression of the target tissue markers *Hnf3β* (*Foxa2*; i–ii), *Shh* (iii–iv), and *Islet-1* ( Isl1;v–vi) appear unaltered in the mutant mice, as determined in horizontal sections. Plane of section is indicated in D. (C) Analysis of *Netrin1* gene expression by in situ hybridization in the hypothalmus of wild-type and *Nestin-Smo* cko mice at E12.5. Expression of the chemoattractant Netrin-1 is unaltered in the mutant mice. Arrows indicate ventral midline/floor plate region where Netrin expression is maximal. Arrowheads indicate weak Netrin expression in the thalamus. Plane of section is indicated in D. (D) Schematic of sagittal section of midbrain at E12.5. Scale bars: (A) 100 µm, (B+C) 200 µm.

To investigate the function of Shh in mDN axonal pathfinding in the intact CNS, we took advantage of conditional mutant mice in which the Shh receptor Smo was inactivated by a transgenic Cre recombinase regulated by the *Nestin* promoter (*Nestin-Smo* cko mice). In these mice Cre-mediated recombination leads to complete inactivation of the Smo allele in the central nervous system by E11.5 [11], [14]. Analysis of lateral dopaminergic fibers that project rostrally to the striatum in the *Nestin-Smo* cko mice at E13.5 revealed normal projections (Figures 3B–3C). In contrast, the medial dopaminergic projections that normally course ventromedially were defective in these mice (Figure 3D, 3E). This was particularly evident in the medial fibers that course most ventrally to extrastriatal targets including the pallidum and the subthalamus, as confirmed by immunostaining for the pallidum precursor marker Nkx2.1 (Figure 4A). Similar to the defects observed at E13.5 in *Nestin-Smo* cko mice, the most medial projections remained defective at a later developmental time point, E15.5 (data not shown). These findings suggest a role for Shh signaling in the ventral targeting of medial dopaminergic axonal projections.

Prior analyses and our expression studies argue strongly against the possibility that the defective mDN axonal targeting in *Nestin-Smo* cko mice is an indirect effect of altered neuron specification of mDNs or target tissue. Patterning and specification of the embryonic ventral midbrain, including mDNs [11], as well as embryonic telencephalic target tissue such as the striatum [14], appear unaltered in *Nestin-Smo* cko mice at the time period (E12.5–E16.5) during which the mDN axonal projections are formed (Figure 3A and Figure S2). The total density of mDN axonal projections appears unaltered in the mutant CNS (data not shown). Genetic fate mapping and conditional knockout analyses have demonstrated that canonical Shh signaling is no longer required in mDNs after E10 [11], [13]. Consistent with this, gene expression of several markers for hypothalamic and forebrain target tissue, including *FoxA2*, *Islet 1*, and *Shh*, appear normal in E12.5 *Nestin-Smo* cko mice mice (Figure 4B). Expression of Netrin1, a chemoattractant present in the ventral midline, is similarly unaltered in the *Nestin-Smo* cko mice (Figure 4C).

**Figure 5:**
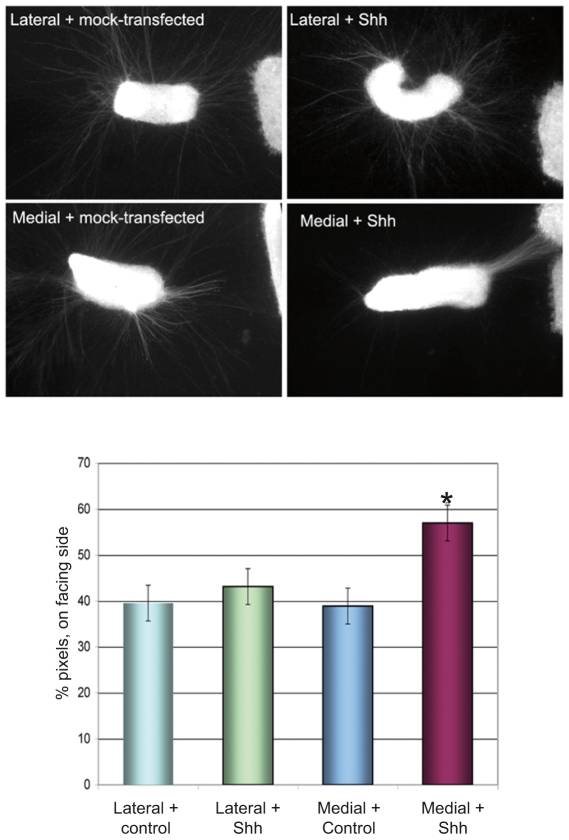
Shh regulation of mDN axonal projections: dissection of signaling interactions. (A) Medial midbrain dopaminergic explants demonstrated increased TH-positive axonal outgrowth from the rostral side (facing the explant) in the presence of a Shh source; in contrast, lateral explants did not appear to be attracted to the Shh source (n>15 explants for each group). The effects of Shh were significant when tested using the two-tailed Student T-test (p<0.05). Scale bar: 100 µm.

We hypothesized that lateral mDN axons, such as those that ascend to the striatum, are intrinsically less sensitive to Shh than the medial projections, and are thus less affected in the *Nestin-Smo* cko mice. To test this, midbrain explants were divided into lateral and medial halves and then tested for chemoattraction to a Shh source (as described above). Axonal projections from medial (but not lateral) midbrain explants displayed unidirectional outgrowth towards the Shh point source (Figure 5, Figure S3). The molecular basis of this differential sensitivity remains to be determined; since the Shh receptor component Smo appears to be expressed evenly in both, medial and lateral mDN (data not shown).

## Discussion

We present evidence that Shh signaling plays an important role in establishing the functional architecture of mDN axonal projections to rostral brain areas. In the absence of Shh signaling, the most medial mDN axons, which are closest to the midline source of Shh in the hypothalamus, fail to course ventrally in their rostral projection. Thus, the gradient of Shh imparts positional information to mDNs that serves to segregate the medial fibers from the lateral fibers. Medial fibers appear to be intrinsically more sensitive to Shh than lateral fibers.

Shh expression starts around E7.5 in the notochord and prechordal mesoderm that underlies the medial neural plate and subsequently induces Shh expression in the ventral midline along the entire neuroaxis of the neural tube. In the midbrain, Shh signaling from the ventral midline is essential for ventral patterning, including the induction of mDNs, but only prior to E10 [10], [11]. Shh expression is maintained in the ventral-medial midbrain and hypothalamus for the subsequent days of embryonic development (Figure 1 and S.B. unpublished data), after mDN induction and differentiation are completed and during the period of mDN axonal outgrowth. Shh in the ventral hypothalamus can therefore act as a local guidance cue for medially projecting dopaminergic axons (Figure 1A).

Several factors have been implicated in mDN axonal pathfinding, and thus we have sought to address the possibility that Shh functions indirectly through these factors to alter mDN pathfinding. Prior studies have presented evidence of a role for Slit-1 and Slit-2 in the chemorepulsion of mDN axons at the midline. Slit2 has been shown to be a chemorepellant for mDNs in explant cultures, and Robo1 and 2, Slit receptors appear to be expressed in mDNs [4]. Furthermore, Shh expression is essential for the expression of Slit1 in the CNS [15]. However, it is unlikely that the phenotype we observe in the absence of Shh signaling is a consequence of deficient Slit expression, as, in fact, mice deficient in Slit1 and Slit2 display the opposite phenotype: ventrally displaced ascending dopaminergic fibers, with a relative expansion of the medial fibers that course ventrally [3]. We therefore hypothesized that Slit signaling may serve to antagonize Shh at the ventral midline and thus refine the axonal targeting of mDN axonal projections. Consistent with this model, Slit2 expression along with Shh in HEK293 effectively suppressed the chemoattractive activity of Shh towards mDN axons in explant cultures (Figure S1).

In vitro studies additionally suggest a role for Netrin signaling in mDN axonal chemoattraction [4]. It is unlikely that Shh functions upstream of Netrin signaling to alter mDN axonal pathfinding, as *Netrin* expression appears unaltered in the conditional *Smo* mutant mice (Figure 4C). We hypothesize that Netrin signaling may serve to refine the synaptic targeting of dopaminergic axons at forebrain regions.

Our data indicate that Shh functions through the Smo receptor as a ventral midline cue for ascending dopaminergic fibers. Furthermore, we find that the other Shh receptor component, Ptc is expressed in the ventral midbrain at this point in development. However, other canonical Shh signaling components, such as Gli1, Gli2, and Gli3, are absent, suggesting a non-canonical pathway. Interestingly, a recent paper demonstrated that Shh guides commissural axons in the spinal cord by stimulating the activity of Src family kinases in a Smo-dependent manner [16]. While this study would indicate a primary role for Smo, prior studies have indicated an additional role for the novel Shh receptors, Boc and Hip, in Shh mediated axonal chemoattraction [17], [9]. It will be of interest to investigate a possible role for these kinases and receptors in mDN axonal targeting.

Further studies are also needed to identify the molecular basis for the differential sensitivity of medial and lateral mDN populations to Shh chemoattraction. We hypothesize that unidentified noncanonical signaling components may be differentially expressed in these cell populations. Alternatively, chemorepulsive signaling pathway components that antagonize the Shh signal, such as Slit pathway components, may be differentially expressed on these cell populations. One limitation to determining differential expression of these signaling components in lateral and medial mDN populations during embryogenesis is the lack of specific markers for embryonic mDN subpopulations.

In conclusion, these studies support a model in which Shh signaling is necessary for the structural and functional diversity of the ascending mDN axonal projections that emanate from the midbrain. Therefore, Shh guidance signaling may play an important role in proper targeting of dopaminergic cells in replacement therapies for Parkinson's disease.

## Methods

### Ethics Statement

All animal studies were performed under an approved IACUC

animal protocol according to the institutional guidelines at Memorial-Sloan Kettering Cancer Center and Columbia University.

### Immunohistochemistry, X-gal staining and in situ hybridization on embryonic sections

*Nestin-Smo* cko mice (*Nestin-Cre; Smo flox/rec*) mice were generated as described [11]. *Ptc-lacZ* [18] mice were described.

Immunohistochemistry and X-gal staining: 12 µm or 30 µm cryosections or 50 µm vibratome sections of 4%PFA fixed mouse embryos were blocked with 5% donkey serum in PBS/1% Triton-X for one hour, stained with sheep anti-TH (1∶500, Pel-Freeze) or rabbit anti-TH (1∶500, Millipore), rabbit anti-Smo (1∶50, Santa Cruz), mouse anti-SHH (1∶100, 5E1 clone, DSHB Iowa) in 5% donkey serum in PBS/1% Triton-X overnight, and labeled with appropriate secondary antibodies (1∶250, Jackson ImmunoResearch) in 5% donkey serum in PBS/1% Triton-X for 2 hours. Sections were imaged using Zeiss LSM510 confocal microscope or a Zeiss Axio observer equipped with ApoTome. The axonal projections of *Nestin-Smo* cko and control mice were assessed by pixel counting TH positive axons projecting towards the LGE in lateral sections and extra-striatal TH positive axons in more medial sections.

In situ hybridization and histology: Embryos or brains were fixed in 4% paraformaldehyde. Paraffin sections (7 µm) were processed for RNA in situ hybridization and histology as described [11].

### Collagen co-cultures

E11.5 mouse embryo midbrains were dissected into bilateral explants of the ventral neuroepithelium containing the dopaminergic midbrain populations (lacking ventral floor plate region). HEK293 cells were transfected with a full-length Shh expression construct (kind gift of T. Jessell) and/or a full-length hSlit2 expression construct (kind gift of M. Tessier-Lavigne) and made into clusters as described previously [19]. Clusters of mock-transfected cells served as controls. Midbrain explants were cultured 100–200 µm from cell clusters in collagen gel as described previously for 3 days. Alternatively ventral midbrain explants were cultured with ventral or dorsal E11.5 rostral midbrain explants. In some experiments the medium was supplemented with cyclopamine (Sigma-Alrich) at 10 nM, with DMSO alone as a control. Co-cultures were fixed in 4% PFA for 2 hours, stained with sheep anti-TH (1∶200, Pel-Freeze) and Cy3 anti-sheep (1∶500, Jackson ImmunoResearch), and imaged using a CCD cooled camera. Images were then analyzed blind using Image Pro software. The explant image was divided into quadrants, and the (TH positive) pixels emanating from each explant side were counted. The number of pixels emanating from the facing side were then expressed as a percentage of the pixels from the facing and the opposing sides. Results were tested for significance using the two-tailed Student T-test.

## Supporting Information

Figure S1 E11.5 bilateral ventral midbrain explants cultures in apposition to mock-transfected, Shh and/or Slit2 transfected HEK293T cells (delineated by dotted lines). Explants demonstrated increased TH positive axonal outgrowth from the rostral side (facing the explant) in the presence of Shh and reduced outgrowth in the presence of Slit2, while in the presence of cells transfected with Shh and Slit2 outgrowth was not significantly different from explants cultured with mock-transfected cells. The axonal extension from the rostral side was quantified using pixel counting software (see Materials and Methods) and expressed as a percentage of total rostral and caudal outgrowth (n>25 explants in each condition). The effects of Shh and Slit2 were significant when tested using the two-tailed Student T-test (p<0.05). Outgrowth from the lateral and caudal sides remained unaffected by the presence of Shh and/or Slit2
(3.42 MB TIF)

Figure S2 (A) Hematoxylin and Eosin staining of E12.5 and E16.5 control and Nestin-Smo cko sagittal brain sections. Midbrain (MB), hypothalamus (Hyp) and forebrain (FB) do not show any obvious changes in the conditional ko. Arrows indicate location of mDN. (HB) Hindbrain, MGE (Medial ganglionic eminence), LGE (lateral ganglionic eminence), Tha (thalamus), POA (preoptic area), RN (red nucleus). (B) Nkx2.1 (red) and TH (Green) immunostaining on E13.5 sagittal sections. TH positive dopaminergic neurons and Nkx2.1 positive areas in the hypothalamus of control and Nestin-Smo cko brains are comparable (outlined). Note that there is background staining on blood vessels. Scale bars: 200 µm
(9.27 MB TIF)

Figure S3 Detailed quantification data for Figure 5.
(0.04 MB PDF)
