# Supplementary figures and images for: Sonic Hedgehog Is a Chemoattractant for Midbrain Dopaminergic Axons
Source: PLoS One. 2009 Sep 23;4(9):e7007. doi: 10.1371/journal.pone.0007007 (PMC2742719; doi:10.1371/journal.pone.0007007)

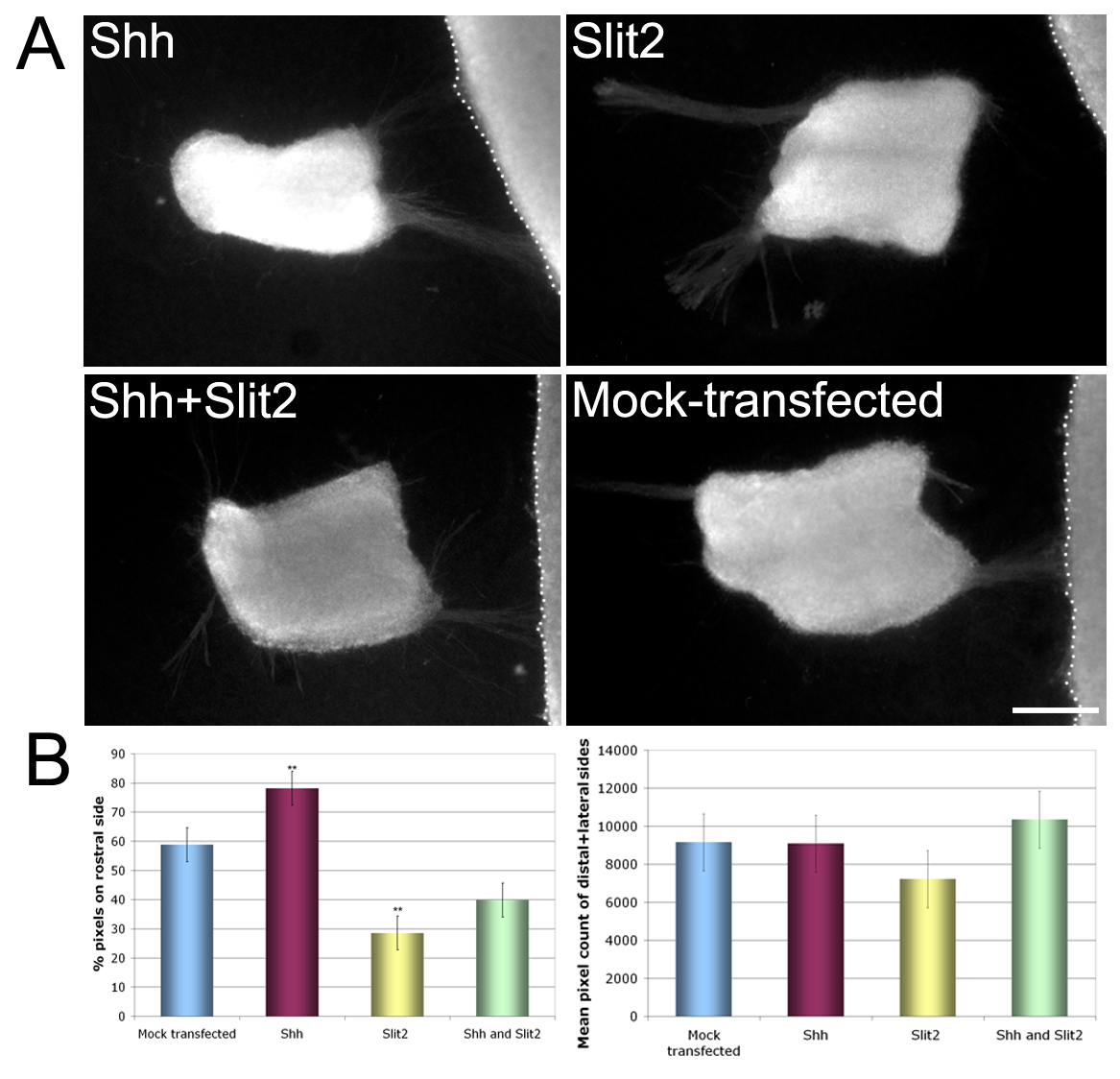

Supplement: Figure S1 — E11.5 bilateral ventral midbrain explants cultures in apposition to mock-transfected, Shh and/or Slit2 transfected HEK293T cells (delineated by dotted lines). Explants demonstrated increased TH positive axonal outgrowth from the rostral side (facing the explant) in the presence of Shh and reduced outgrowth in the presence of Slit2, while in the presence of cells transfected with Shh and Slit2 outgrowth was not significantly different from explants cultured with mock-transfected cells. The axonal extension from the rostral side was quantified using pixel counting software (see Materials and Methods) and expressed as a percentage of total rostral and caudal outgrowth (n>25 explants in each condition). The effects of Shh and Slit2 were significant when tested using the two-tailed Student T-test (p<0.05). Outgrowth from the lateral and caudal sides remained unaffected by the presence of Shh and/or Slit2 (3.42 MB TIF) [file pone.0007007.s001.tif]

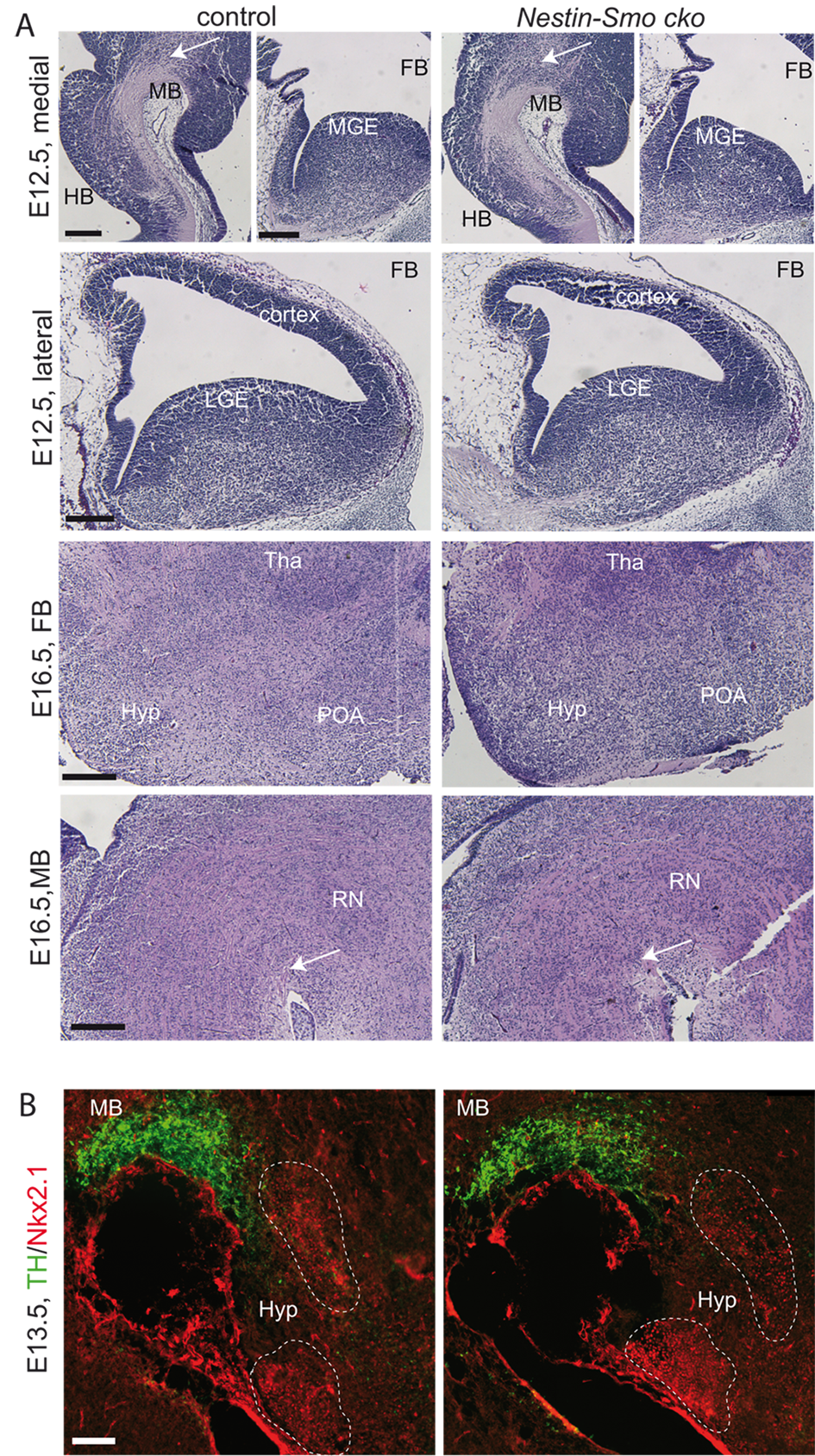

Supplement: Figure S2 — (A) Hematoxylin and Eosin staining of E12.5 and E16.5 control and Nestin-Smo cko sagittal brain sections. Midbrain (MB), hypothalamus (Hyp) and forebrain (FB) do not show any obvious changes in the conditional ko. Arrows indicate location of mDN. (HB) Hindbrain, MGE (Medial ganglionic eminence), LGE (lateral ganglionic eminence), Tha (thalamus), POA (preoptic area), RN (red nucleus). (B) Nkx2.1 (red) and TH (Green) immunostaining on E13.5 sagittal sections. TH positive dopaminergic neurons and Nkx2.1 positive areas in the hypothalamus of control and Nestin-Smo cko brains are comparable (outlined). Note that there is background staining on blood vessels. Scale bars: 200 µm (9.27 MB TIF) [file pone.0007007.s002.tif]
