# Supplementary material for: Sonic Hedgehog Is a Chemoattractant for Midbrain Dopaminergic Axons
Source: PLoS One. 2009 Sep 23;4(9):e7007. doi: 10.1371/journal.pone.0007007 (PMC2742719; doi:10.1371/journal.pone.0007007)
Supplement: Figure S3 — Detailed quantification data for Figure 5. (0.04 MB PDF) [file pone.0007007.s003.pdf]

dorsal vs ventral shh vs control expt **Supplementary Figure 3**

Dorsal control

	facing	opposing	left	right	f/f+o*100
1	3441	8567	14790	1356	28,6559
2	1933	4603	4770	1928	29,57466
3	2271	4387	6049	4764	34,10934
4	1528	3263	2400	1508	31,89313
5	3070	3024	1877	1238	50,37742
7	668	3553	2249	1537	15,82563
8	1884	3340	5526	2187	36,06432
9	4844	4745	1769	5277	50,51622
10	5060	19759	3746	4032	20,38761
11	13695	1163	4851	1603	92,17257
12	9244	9759	7543	9097	48,64495
13	5628	9918	14550	4651	36,20224
n	12	root n	3,464102		
Av	4438,833	6340,083	5843,333	3264,833	39,53533
st dev	3739,2	5071,566	4497,718	2380,882	19,88412
st error	1079,414	1464,035	1298,379	687,3015	5,74005
st error/2	539,707	732,0175	649,1896	343,6507	2,870025

Dorsal Shh

	facing	opposing	left	right	f/f+o*100
1	7830	7477	17394	1685	51,15307
2	6806	5655	13047	4763	54,61841
3	8379	17913	10344	7371	31,86901
4	7440	9813	20591	7158	43,12294
5	14251	47430	18600	1566	23,10436
7	8962	22323	21808	8030	28,64632
8	10163	7612	4822	10619	57,17581
9	17278	6739	9657	12663	71,94071
10	7489	12984	14396	10784	36,57989
12	1823	3604	6214	1186	33,5913
14	7430	5738	12352	3348	56,42467
15	5539	42642	5484	5896	11,49623
16	21220	14794	11003	18218	58,92153
17	6707	3326	5668	1873	66,8494
18	3416	5109	6408	2600	40,07038
19	5393	12788	10705	2276	29,66283
20	6193	10107	8651	16927	37,99387
n	17	root n	4,123106		
Av	8607	13885,53	11596,71	6880,176	43,13063
st dev	4865,191	12828,55	5407,151	5384,911	16,39689
st error	1179,982	3111,38	1311,427	1306,033	3,976831
st error/2	589,9911	1555,69	655,7134	653,0164	1,988415

Ventral control

	facing	opposing	left	right	f/f+o*100
5	22075	16080	12646	11983	57,85611
6	2962	12002	3889	5335	19,79417
7	15187	20559	585	889	42,48587
8	8532	44239	6021	1000	16,16797
9	8017	4556	15640	13109	63,76362
10	6541	5428	839	4073	54,64951
11	2242	10480	13006	1439	17,62302

n	7	root n	2,645751		
Av	9365,143	16192	7518	5404	38,90575
st dev	7043,452	13584,06	6197,682	5162,82	20,708
st error	2662,175	5134,293	2342,504	1951,362	7,826888
st error/2	1331,087	2567,147	1171,252	975,6812	3,913444

Ventral Shh

	facing	opposing	left	right	f/f+o*100
1	5199	5720	3438	12678	47,61425
5	8632	10046	1891	1134	46,2148
8	15309	20829	6480	6249	42,36261
9	1879	4874	1457	2422	27,82467
10	12128	1732	17748	4407	87,50361
12	13225	9478	4819	8174	58,25221
13	16957	4594	1462	521	78,68312
15	1823	2244	6252	281	44,82419
16	7576	3503	6479	1767	68,38162
17	14897	27453	771	884	35,17591
18	8416	4960	13905	1862	62,91866
21	5150	7734	1251	2615	39,97206
22	6316	1947	931	594	76,43713
24	2662	903	881	959	74,67041
26	11121	10827	3535	189	50,66976
27	25411	11090	1464	1818	69,61727
n	16	root n	4		
Av	9793,813	7995,875	4547,75	2909,625	56,94514
st dev	6380,338	7222,395	4911,239	3434,023	17,58861
st error	1595,085	1805,599	1227,81	858,5058	4,397153
st error/2	797,5423	902,7994	613,9049	429,2529	2,198577
